# KLF14 targets ITGB1 to inhibit the progression of cervical cancer via the PI3K/AKT signalling pathway

**DOI:** 10.1007/s12672-022-00494-1

**Published:** 2022-05-16

**Authors:** Xinran Lyu, Xuchao Ding, Hui Ye, Rong Guo, Minhang Wu, Lili Cao

**Affiliations:** 1 Oncology Department, The First Affiliated Hospital of Shandong First Medical University & Shandong Provincial Qianfoshan Hospital, Jinan, 250014 China; 2 Oncology Department, Shandong Provincial Qianfoshan Hospital, School of Medicine, Shandong University, Jinan, 250014 China; 3 Shandong University of Traditional Chinese Medicine, Jinan, 250014 China; 4 Shandong Provincial Key Laboratory for Rheumatic Disease and Translational Medicine, Jinan, 250014 China

**Keywords:** Cervical cancer, KLF14, ITGB1, PI3K/AKT signalling pathway, Apoptosis

## Abstract

Our study aimed to determine whether Krüppel-like factor 14 (KLF14) inhibits the proliferation and promotes the apoptosis of cervical cancer cells through integrin β1 (ITGB1). Immunohistochemistry was performed to determine the expression of KLF14. The effect of KLF14 on the proliferation of cervical cancer cells was verified by Cell Counting Kit-8 (CCK-8) assays, colony formation assays and in vivo experiments. The effect of KLF14 on cervical cancer cell apoptosis was detected by flow cytometry. The targeting relationship between KLF14 and ITGB1 was evaluated by Western blotting and a dual-luciferase reporter assay. Moreover, Flow cytometry was performed to verify the relationship between KLF14 and ITGB1 on the apoptosis of cervical cancer cells. Additionally, Western blot analysis was performed to investigate the relationship between KLF14 and ITGB1 on the expression of downstream related molecules. As a result, the expression of KLF14 in cervical cancer tissues was lower than that in paracancerous tissues. KLF14 inhibited proliferation and promoted apoptosis in cervical cancer cells. Mechanistically, ITGB1 expression was significantly downregulated in KLF14-overexpressing cervical cancer cells. At the same time, we found that the effects of KLF14 and ITGB1 on apoptosis of cervical cancer cells could be mutually affected. KLF14 directly targeted ITGB1 to regulate its downstream PI3K/AKT signalling pathway. In summary, KLF14 inhibits the progression of cervical cancer by targeting ITGB1 via the PI3K/AKT signalling pathway.

**Supplementary Information:**

The online version contains supplementary material available at 10.1007/s12672-022-00494-1.

## Background

The Global Cancer Observatory reported that cervical cancer was the fourth most common cancer in women worldwide in 2020 [1]. The incidence and mortality of cervical cancer are especially high in transitioning countries [2]. At present, human papillomavirus (HPV) infection is the most important cause of cervical cancer and the occurrence of cervical cancer is closely related. More than 99% of cervical cancers are due to persistent infection with high-risk human papillomaviruses (HR-HPVs), especially HPV-16 and HPV-18. The occurrence of cervical cancer caused by HPV infection is related to E5, E6 and E7 proteins, which are interrelated with many processes in life activities, resulting in increased cell proliferation, blocked differentiation, reduced apoptosis and even gene mutation, thus promoting the occurrence and development of cervical cancer [3, 4]. With the improvement of treatment methods and the advent of the human papillomavirus (HPV) vaccine, the treatment and prevention of cervical cancer have greatly improved [5]. However, tumour metastasis and recurrence may occur due to differences in tumour grade and biological behaviour. Especially for patients in the middle and advanced stages, the current treatment methods are relatively limited, the effect of radiotherapy and chemotherapy is not ideal, and the prognosis of cervical cancer patients is poor, posing a serious threat to women’s health [6].

Krüppel-like factor 14 (KLF14), a member of the SP/KLF14 gene family, is a transcription factor involved in the regulation of many physiological and pathological processes [7, 8]. Most recent studies have focused on the role of KLF14 in cell metabolism [9]. Some studies have shown that KLF14 is closely related to type 2 diabetes and can improve insulin sensitivity and insulin resistance [10, 11]. In addition, KLF14 is associated with a number of cardiovascular diseases and prevents and reduces atherosclerosis by increasing cholesterol excretion and reducing inflammation [12]. Recently, KLF14 has also been found to be associated with the occurrence and progression of tumours. By reducing LDHB, glycolysis can be regulated to cut off the supply of cancer cells, thus inhibiting the progression of colon cancer [13]. KLF14 is regulated as a target gene of lncRNA DGCR5 to inhibit the progression of hepatocellular carcinoma (HCC) [14]. At present, there are relatively few studies on the role of KLF14 in cervical cancer. Integrins are heterodimeric cell surface receptors and play an important role in cell differentiation, migration, proliferation, adhesion and tumour progression [15, 16]. With increased research on the integrin signalling pathway and related molecules [17], integrins have become a hot target in antitumour therapy. Integrin β1 (ITGB1), a member of the integrin (ITG) family, regulates tumour invasion, migration, and apoptosis [18, 19]. On this basis, we considered whether KLF14 might be associated with ITGB1, thereby affecting the development of cervical cancer.

PI3K/AKT signaling pathway is a crucial signal transduction pathway in cells, participating in many important life processes such as cell proliferation, apoptosis and angiogenesis, and its disorder leads to diseases such as diabetes, cardiovascular diseases and cancer. At present, the relationship between THE PI3K/AKT signaling pathway and the occurrence and development of tumors is a research hotspot. Most cancers are closely associated with this pathway, so finding molecules or drugs that regulate this pathway has become the research direction of cancer treatment [20, 21]. Studies have shown that KLF14 may increase insulin sensitivity by activating the PI3K/AKT signaling pathway, thereby affecting glucose metabolism [22]. At present, the relationship between KLF14 and PI3K/AKT signaling pathway is rarely studied in tumors. ITGB1 is closely related to PI3K/AKT signaling pathway, and has been widely studied in tumors, including hepatocellular carcinoma and cervical cancer [23, 24]. Therefore, we considered whether KLF14, ITGB1 and PI3K/AKT signaling pathways are correlated to affect the progression of cervical cancer.

In this study, we investigated whether KLF14 can inhibit the proliferation and promote the apoptosis of cervical cancer cells. Moreover, we further explored whether KLF14 regulates ITGB1 and plays an inhibitory role in the progression of cervical cancer by regulating the PI3K/AKT signalling pathway.

## Materials and methods

### Cell culture

Human cervical cancer cells, including SiHa and HeLa cells, were used in this study; HeLa cells were purchased from the American Type Culture Collection (ATCC, USA), and SiHa cells were purchased from the Chinese Tissue Culture Collections (CTCC, China). These cells were cultured in DMEM (Gibco, USA) containing 10% foetal bovine serum (FBS, Gibco, USA) and 1% penicillin–streptomycin and incubated in a constant-temperature incubator at 37 °C with 5% CO_2_.

### Cell transfection

Construction of KLF14 overexpression lentivirus stably transfected cell lines: KLF14 overexpression lentivirus and its negative control lentivirus were constructed by GeneChem (Shanghai, China). KLF14 overexpression lentivirus and its negative control lentivirus were transfected into SiHa or HeLa cells. The cells were screened by treatment with 2.5 μg/mL puromycin, and the expression level of KLF14 was verified by Western blotting. In subsequent experiments, cells were precultured with doxorubicin (DOX, 5 µg/ml)-containing medium for 48 h to induce KLF14 expression.

Construction of ITGB1 overexpression lentivirus stably transfected cell lines: ITGB1 overexpression lentivirus and its negative control lentivirus were constructed by GeneChem (Shanghai, China). The ITGB1 overexpression lentivirus and its negative control lentivirus were transfected into SiHa cells. The cells were screened by treatment with 2.5 μg/mL puromycin, and the expression level of ITGB1 was verified by Western blotting.

Cotransfection of cells with lentiviruses targeting ITGB1 and KLF14: A. KLF14 overexpression lentivirus and its negative control lentivirus were transfected into SiHa cells transfected with Lv-ITGB1. B. The ITGB1 overexpression lentivirus and its negative control lentivirus were transfected into SiHa cells transfected with Lv-KLF14. The expression levels of ITGB1 and KLF14 were detected by Western blotting after puromycin screening.

### Immunohistochemistry

A human cervical cancer tissue microarray (OD-CT-RpUtr03-004) was purchased from Shanghai Xinchao Biotechnology Co., Ltd. The tissue chip contained 31 cervical cancer tissues and their paired adjacent tissues, along with clinical data including age, sex, tumour-node-metastasis (TNM) stage and clinical stage for the corresponding patients. Specific patient information can be found in the Additional file 1. The tissue chip was baked, dewaxed and hydrated with ethanol, followed by antigen repair and endogenous peroxidase blocking. The tissue chip was covered with a 1:500 dilution of KLF14 primary antibody (1:500, Sigma–Aldrich, #HPA044729) and incubated overnight at 4 °C. The next day, the chip was incubated with secondary antibody for 30 min at room temperature. The sections were stained with DAB and haematoxylin, dehydrated, cleared and sealed. Finally, the images were observed under a microscope, photographed and graded.

### Cell counting kit-8 (CCK-8) assay

Cells of the Lv-control group and Lv-KLF14 group were seeded in 96-well plates at a density of 5000 cells per well, and the blank group with culture medium was set. Each condition was plated in 6 replicate wells. After approximately 4–6 h, when the cells were completely attached to the well, the absorbance value of each well at 450 nm was detected as the cell state at 0 h. Then, all the medium in the well was removed and replaced with DOX-containing medium, and the cells were placed into an incubator for culture. After 24 h or 48 h, CCK-8 reagent (#CK04, Dojindo) was added to each well in a dark environment, and the cells were cultured immediately in an incubator for 2 h. The absorbance value at 450 nm was assessed to determine the cell state at 24 or 48 h.

### Colony formation assay

Cells of the Lv-control group and Lv-KLF14 group were evenly seeded on petri dishes at a density of 2000 cells per dish and cultured in medium containing DOX for approximately 14 days. When obvious colonies appeared, the medium in the dish was discarded, and the cells were washed with PBS, fixed with methanol for 15–20 min, and then stained with 1% crystal violet for 20 min. After washing with PBS, the cells were photographed and counted.

### Flow cytometry

The cells (1 × 10^6^) were cultured in six-well plates, three repeated wells were set for each group. ITGB1-overexpressing cells were cultured in standard medium, while KLF14-overexpressing cells were cultured in medium containing DOX (5 µg/ml). After overnight incubation in the incubator, the original medium was discarded, and the cells were digested with trypsin after rinsing with PBS and collected into EP tubes. Centrifugation was conducted at 1500 RPM for 5 min, and the cell precipitate was retained. Then, the instructions of the APC Annexin V Apoptosis Detection Kit with PI (#640932, BioLegend, USA) were followed. First, cell resuspension with 400 µl Annexin V Binding Buffer. In the resuspended cell suspension, 100 µl was taken in a new EP tube, and each tube was well labeled. Each tube was supplemented with 5 µl APC Annexin V and 10 µl Propidium Iodide Solution, mixed with gentle vortex oscillation, and incubated at 25 ℃ for 15–20 min. After incubation, 400 µl Annexin V Binding Buffer was added to each tube and mixed. The whole process needs to be protected from light, and to be conducted in a dark environment. Finally, the apoptosis rate was detected by flow cytometry. The experiment was repeated three times.

### Establishment of a subcutaneous tumorigenesis model in nude mice

BALB/C female nude mice aged 4–5 weeks were purchased from Vital River Lab Animal Technology Co., Ltd. We established six groups and a specific treatment scheme. First, the injection scheme included six nude mice in total, and the left and right sides of each nude mouse were compared. SiHa cells (5 × 10^6^ cells/group) of the Lv-KLF14 group were injected subcutaneously into the axilla of the right upper limb of nude mice with 200 μl PBS suspension as the positive control. SiHa cells (5 × 10^6^ cells/group) of the Lv-control group were also injected subcutaneously into the left upper arm axilla of nude mice with 200 μl PBS suspension as the negative control group. Next, DOX, as an inducer, induced KLF14 expression in the Lv-KLF14 group, while KLF14 was not expressed in the Lv-control group, allowing for comparison. We then set up an induction scheme. Before the injection, SiHa cells of the Lv-KLF14 group and Lv-control group were cultured in medium containing DOX (5 µg/ml) for 48 h. After the injection, the nude mice were fed water with DOX (1 mg/ml) for 1 week. After that, DOX-containing water was fed intermittently. We have made a model diagram and table for the treatment scheme, which can be seen in Additional file 2. Subcutaneous tumour growth was observed every 10 days, and the tumour volume was recorded. Approximately 2 months later, the nude mice were sacrificed, and the tumours that had formed under the skin were removed, photographed, weighed and frozen at − 80 °C. The tumour tissue proteins of the Lv-control group and Lv-KLF14 group were extracted for Western blotting, and the expression of KLF14 (1:500, #HPA044729, Sigma–Aldrich), ITGB1 (1:1000, #4706, CST), AKT (1:400, #4691, CST), p-AKT (1:400, #4060, CST) and cleaved caspase-3 (1:400, #9661S, CST) in tumour tissues was detected. This experiment was approved by the Ethics Committee of the First Affiliated Hospital of Shandong First Medical University (SYDWLS2020016), animal experiments strictly complied with national laws, regulations and standards related to experimental animals, including the Regulations on the Management of Experimental Animals and the Guidelines for Ethical Review of Experimental Animal Welfare, and referred to the consensus of relevant guidelines on animal experimental research reports in international biomedical journals (ARRIVE Guidelines).

### Western blotting

RIPA lysis buffer was used to extract cell protein samples. The cell proteins were separated using 10% SDS–PAGE and then transferred to PVDF membranes. After transfer, the cells were blocked with 5% skim milk powder for 1 h. The primary antibodies were as follows: FLAG (1:1000, #14793S, CST), ITGB1 (1:1000, #4706, CST), KLF14 (1:500, #HPA044729, Sigma–Aldrich), cleaved caspase-3 (1:400, #9661S, CST), AKT (1:400, #4691, CST), p-AKT (1:400, #4060, CST), PI3K (1:250, #4249, CST), Bax (1:400, #89744, CST), and PDK1 (1:400, #5662, CST). The secondary antibody was horseradish peroxidase-labelled goat anti-rabbit antibody or anti-mouse antibody. The reference antibody was beta-actin (1:1000, #AF7018, Affinity). Finally, the protein expression level was observed on a chemiluminescence imager with an enhanced chemiluminescence (ECL) kit (Millipore, USA). The greyscale values of the protein bands were analysed by ImageJ software.

(Western blotting in Figs. 2 and 5 were loaded with 210 μg of protein per lane in each gel. For other Western blotting, each gel was loaded with 315 μg of protein per lane).

### Dual-luciferase reporter assay

The KLF14 plasmid and promoter ITGB1 were constructed by GeneChem (Shanghai, China). HEK-293 T cells were seeded into 24-well plates for 24 h before transfection. The cells were cotransfected using PolyJet (SignaGen) with KLF14 plasmid and promoter ITGB1 as the positive group. The cells were cotransfected using PolyJet with KLF14 empty plasmid and promoter ITGB1 as the negative group. Twenty-four hours after transfection, the dual-luciferase reporter assay system (Promega) was used to detect firefly and Renilla luciferase activity according to the manufacturer’s instructions. The relative luciferase activity was calculated and quantified.

### Statistical analysis

Data analysis was performed with GraphPad Prism 8.0. The differences between groups were analysed by Student’s t test, one-way ANOVA and multiple t test. The data were considered statistically significant when the P value was less than 0.05.

## Results

### KLF14 is expressed at low levels in cervical cancer tissues

**Fig. 1:**
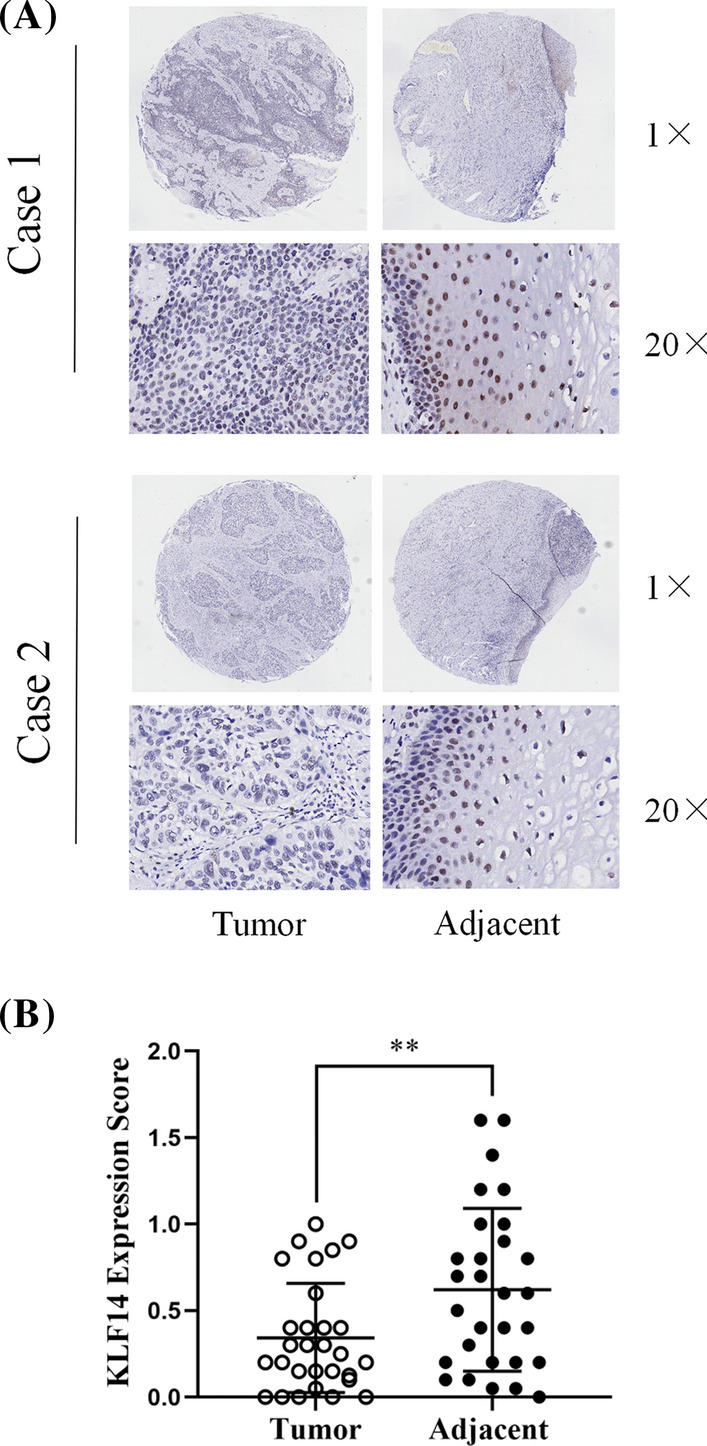
KLF14 is expressed at low levels in cervical cancer tissues. **A** The immunohistochemical results showed that the expression level of KLF14 in cervical cancer tissues was lower than that in adjacent tissues. **B** We scored each tissue point (total score = staining intensity × positive rate). Quantitative statistics of immunohistochemical results. (**P < 0.01)

To assess the expression level of KLF14 in cervical cancer tissues, we performed immunohistochemistry analysis of a tissue microarray containing 31 cervical cancer tissues and their adjacent tissues. We scored each tissue point (total score = staining intensity × positive rate). The KLF14 expression level in cervical cancer tissues was lower than that in adjacent tissues in 22 cases and higher than that in adjacent tissues in 7 cases. There were 2 cases of delamination. This indicated that in most cases, KLF14 expression was low in cervical cancer tissues, but high in paracancer tissues. Paired T test was performed for statistical analysis, and the difference was statistically significant. Therefore, the KLF14 expression level in cervical cancer tissues was lower than that in adjacent tissues (Fig. 1A, B).

### KLF14 inhibited cervical cancer cell proliferation in vitro

**Fig. 2:**
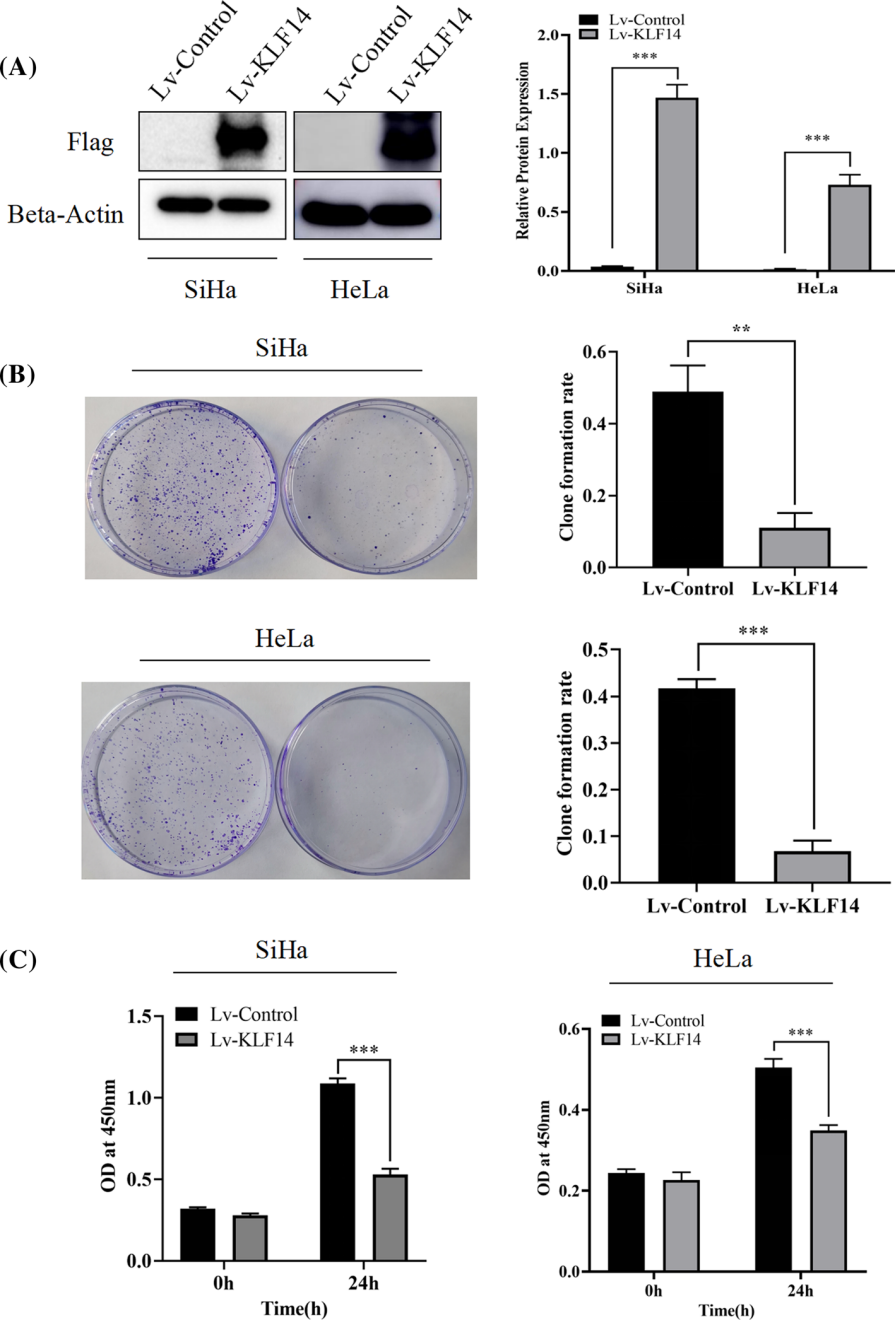
KLF14 inhibited cervical cancer cell proliferation in vitro. **A** After transfection with Lv-KLF14, the Western blot results showed that the expression of KLF14 was significantly increased in SiHa and HeLa cells. **B** The colony formation assay verified that the colony formation rate of the Lv-KLF14 group was lower than that of the Lv-control group. **C** The CCK-8 assay demonstrated that the proliferation of the Lv-KLF14 group was lower than that of the Lv-control group within 24 h. These results suggest that KLF14 has an inhibitory effect on the proliferation of cervical cancer SiHa cells and HeLa cells. (**P < 0.01, ***P < 0.001)

For the following experiments, SiHa and HeLa cells were transfected with Lv-control and Lv-KLF14. Western blotting was used to verify the KLF14 transfection efficiency. In both SiHa and HeLa cells, the expression of KLF14 in the Lv-KLF14 group was significantly higher than that in the Lv-control group (Fig. 2A). In the colony formation assay, SiHa and HeLa Lv-KLF14 cells showed lower colony formation rates than Lv-control cells during the same time period (Fig. 2B). In the CCK-8 assay, the absorbance value at 450 nm of SiHa and HeLa cells in the Lv-KLF14 group was lower than that in the Lv-control group after 24 h. Therefore, the proliferation rates of SiHa and HeLa cells in the Lv-KLF14 group were lower than those in the Lv-control group during the same time period (Fig. 2C). In general, we believe that KLF14 has an inhibitory effect on the proliferation of cervical cancer cells.

### KLF14 inhibited the progression of cervical cancer in vivo

**Fig. 3:**
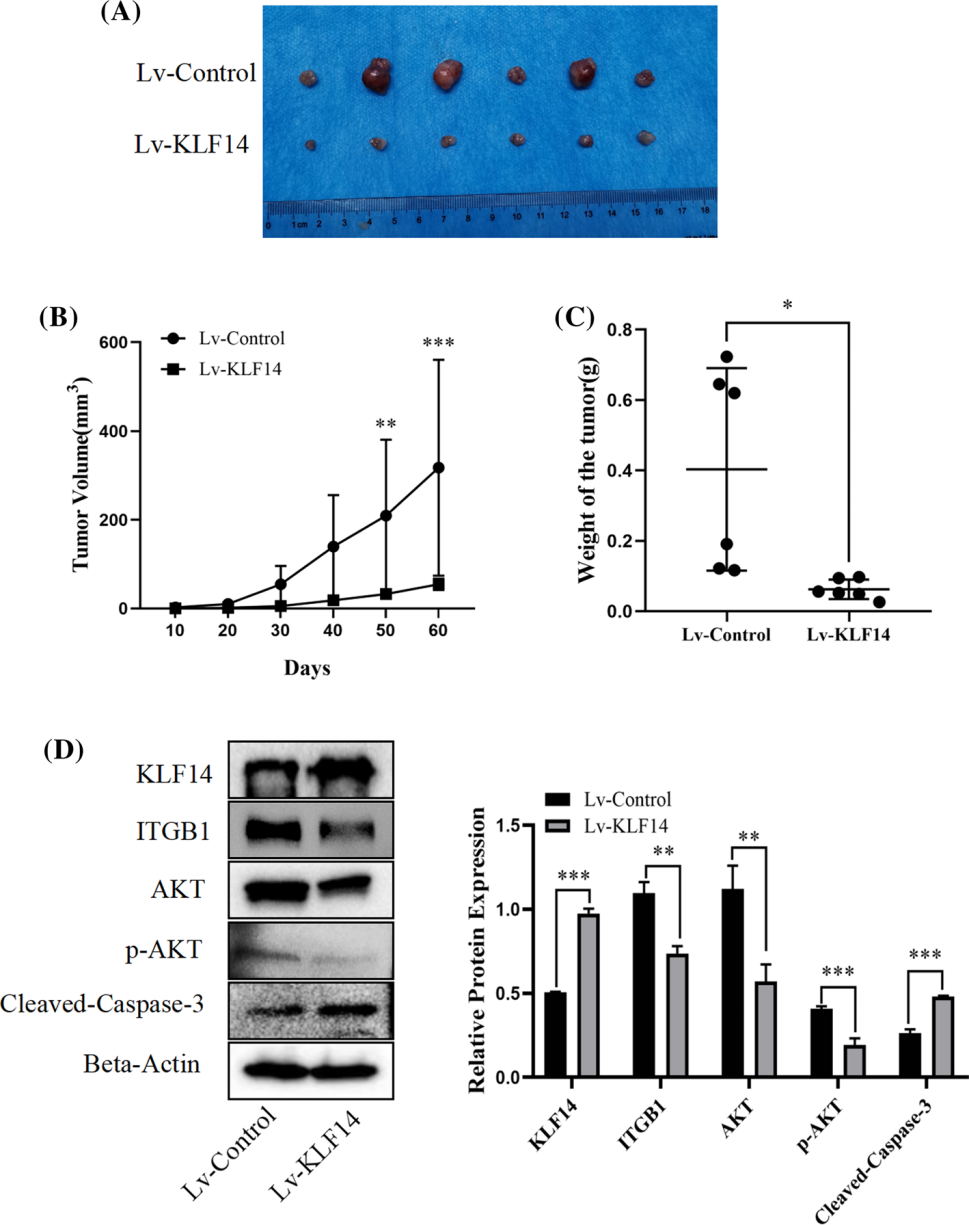
KLF14 inhibited the progression of cervical cancer in vivo. **A** First, six groups were set up. The injection scheme was as follows: six nude mice were used, and the left and right sides of each nude mouse were compared. SiHa cells (5 × 10^6^ cells/group) of the Lv-KLF14 group were injected subcutaneously into the axilla of the right upper limb of nude mice with 200 μl PBS suspension as the positive control. The Lv-control group was injected subcutaneously into the left upper arm axilla of nude mice with 200 μl PBS suspension as the negative control. Next, DOX, as an inducer, induced KLF14 expression in the Lv-KLF14 group, while KLF14 was not expressed in the Lv-control group, allowing for comparison. The induction scheme was as follows: before the injection, SiHa cells of the Lv-KLF14 group and Lv-control group were cultured in medium containing DOX (5 µg/ml) for 48 h. After the injection, the nude mice were fed water with DOX (1 mg/ml) for 1 week. After that, DOX-containing water was provided intermittently. Two months later, the nude mice were sacrificed, and the size of subcutaneous tumours in the Lv-KLF14 group was smaller than that in the Lv-control group. **B** Tumour growth was recorded every 10 days, and tumour volume was calculated using the formula V = (larger diameter) × (smaller diameter)^2^/2. The results showed that tumour growth was faster in the Lv-control group than in the Lv-KLF14 group. **C** The weight of subcutaneous tumours in the Lv-KLF14 group was lower than that in the Lv-control group. **D** According to the Western blot results, the expression of KLF14 and cleaved caspase-3 was upregulated and the expression of ITGB1 and p-AKT was downregulated in the Lv-KLF14 group compared with the Lv-control group in tumour tissues. (*P < 0.05, **P < 0.01, ***P < 0.001)

SiHa cells from the Lv-KLF14 group and Lv-control group were suspended in 200 μl PBS and injected subcutaneously to establish a subcutaneous tumorigenesis model in nude mice. The results showed that tumours in the Lv-KLF14 group were smaller than those in the Lv-control group (Fig. 3A). During the experiment, tumour size was recorded every 10 days, tumour volume was calculated using the formula V = (larger diameter) × (smaller diameter)^2^/2, and the tumour volume growth curve was plotted. The tumours in the Lv-control group grew faster than those in the Lv-KLF14 group. In the Lv-KLF14 group, tumour growth was inhibited (Fig. 3B). The tumour weight in the Lv-KLF14 group was significantly lower than that in the Lv-control group (Fig. 3C). For further verification, we explored the expression of KLF14 and other related molecules in tumour tissues. According to the Western blot results, the expression of KLF14 and cleaved caspase-3 was upregulated, and the expression of ITGB1 and p-AKT was downregulated in the Lv-KLF14 group compared with the Lv-control group (Fig. 3D).

### KLF14 promotes the apoptosis of cervical cancer cells

**Fig. 4:**
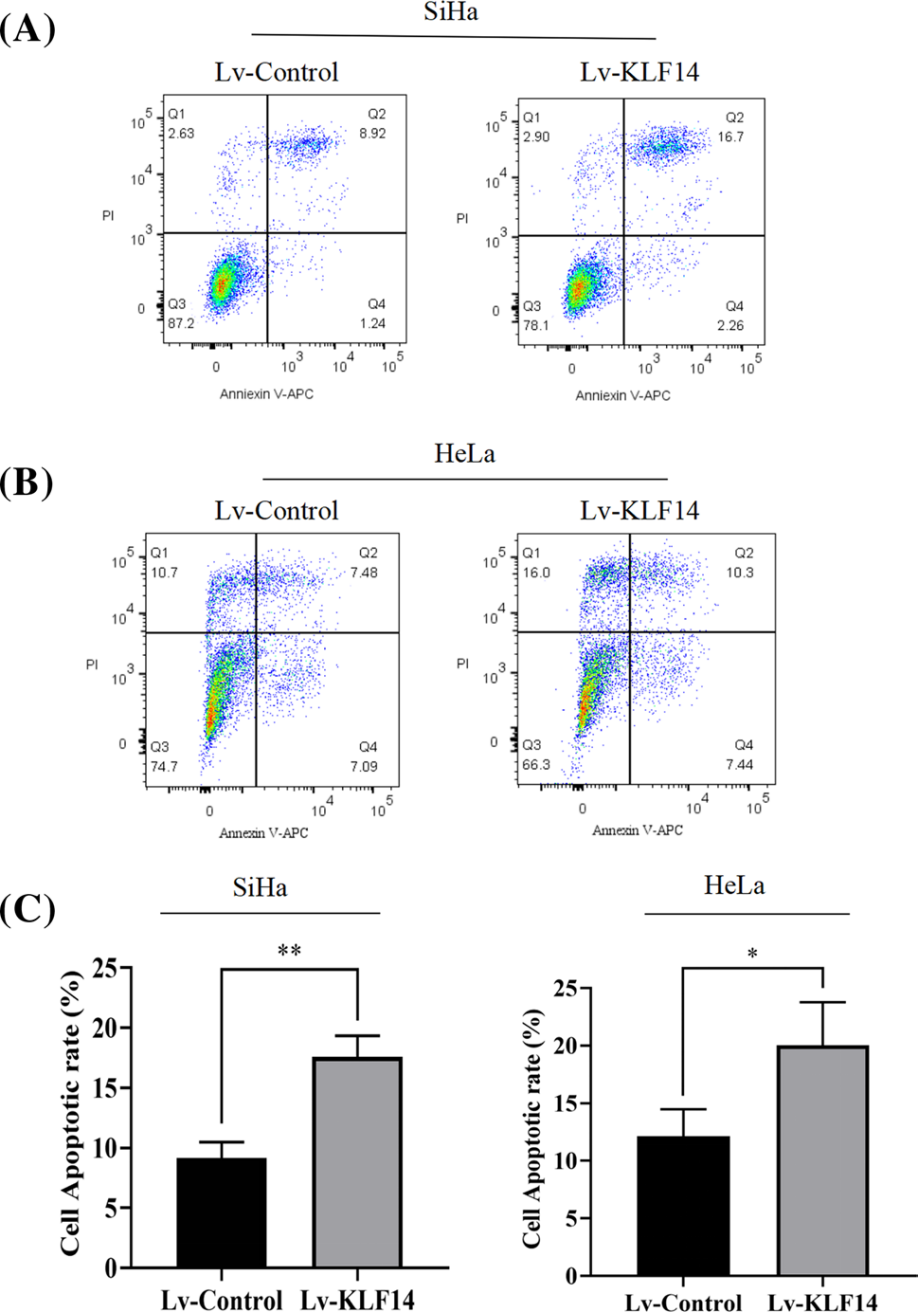
KLF14 promotes cervical cancer cell apoptosis. **A** Flow cytometry was performed to detect apoptosis, and the results showed that the apoptosis rate of SiHa cells in the Lv-KLF14 group was higher than that of SiHa cells in the Lv-control group. **B** The apoptosis rate of HeLa cells in the Lv-KLF14 group was higher than that of HeLa cells in the Lv-control group. **C** Quantitative statistics of the apoptosis rate of SiHa cells and HeLa cells. (*P < 0.05, **P < 0.01)

To verify the effect of KLF14 on the apoptosis of cervical cancer cells, flow cytometry was performed on cervical cancer cells from the Lv-control group and Lv-KLF14 group, including SiHa cells and HeLa cells. The results showed that the apoptosis rate of both SiHa cells and HeLa cells in the Lv-KLF14 group was higher than that in the Lv-control group (Fig. 4A–C), suggesting that KLF14 can promote the apoptosis of cervical cancer cells.

### KLF14 regulates ITGB1 and affects the apoptosis of cervical cancer cells

**Fig. 5:**
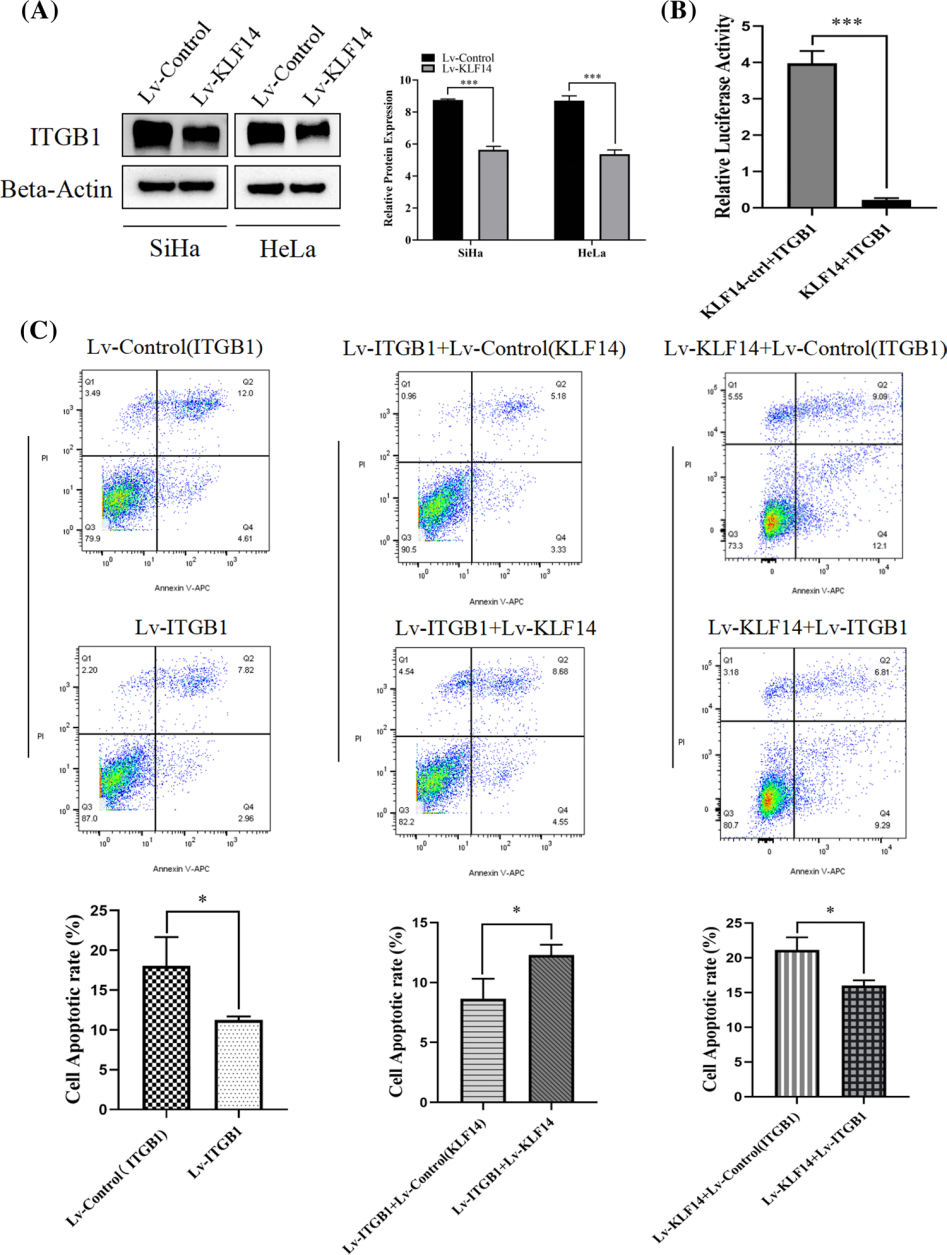
KLF14 regulates ITGB1 and affects cervical cancer cell apoptosis. **A** The Western blot results showed that the ITGB1 expression level in the Lv-KLF14 group was lower than that in the Lv-control group. **B** The dual-luciferase reporter assay results showed that KLF14 directly targeted ITGB1 and downregulated ITGB1 expression. **C** Six groups were established: Lv-control (ITGB1), Lv-ITGB1, Lv-ITGB1 + Lv-control (KLF14), Lv-ITGB1 + Lv-KLF14, Lv-KLF14 + Lv-control (ITGB1) and Lv-KLF14 + Lv-ITGB1. [**A** Lv-ITGB1 + Lv-control (KLF14) and Lv-ITGB1 + Lv-KLF14, KLF14 overexpression lentivirus and its negative control lentivirus were transfected into SiHa cells transfected with Lv-ITGB1. **B** Lv-KLF14 + Lv-control (ITGB1) and Lv-KLF14 + Lv-ITGB1, ITGB1 overexpression lentivirus and its negative control lentivirus were transfected into SiHa cells transfected with Lv-KLF14]. Flow cytometry confirmed that the apoptosis rate of SiHa cervical cancer cells in the Lv-ITGB1 group was lower than that in the Lv-control (ITGB1) group. The apoptosis rate of SiHa cells in the Lv-ITGB1 + Lv-KLF14 group was higher than that in the Lv-ITGB1 + Lv-control (KLF14) group. The apoptosis rate of cervical cancer cells in the Lv-KLF14 + Lv-ITGB1 group was lower than that of cervical cancer cells in the Lv-KLF14 + Lv-control (ITGB1) group. (*P < 0.05, ***P < 0.001)

ITGB1 is a member of the ITG family that is closely related to apoptosis. Therefore, we considered that KLF14 might associate with ITGB1 to promote the apoptosis of cervical cancer cells, thereby inhibiting the development of cervical cancer. Western blotting was performed to verify the expression level of ITGB1 in the Lv-control group and Lv-KLF14 group, and the results showed that the expression level of ITGB1 in the Lv-KLF14 group was lower than that in the Lv-control group (Fig. 5A). A dual-luciferase reporter assay was conducted to detect the targeting relationship between KLF14 and ITGB1, and the results showed that KLF14 directly targeted ITGB1 and downregulated ITGB1 expression (Fig. 5B). In addition, six groups were established: Lv-control (ITGB1), Lv-ITGB1, Lv-ITGB1 + Lv-control (KLF14), Lv-ITGB1 + Lv-KLF14, Lv-KLF14 + Lv-control (ITGB1) and Lv-KLF14 + Lv-ITGB1. For the Lv-ITGB1 + Lv-control (KLF14) and Lv-ITGB1 + Lv-KLF14 groups, the KLF14 overexpression lentivirus and its negative control lentivirus were transfected into SiHa cells transfected with Lv-ITGB1. For the Lv-KLF14 + Lv-control (ITGB1) and Lv-KLF14 + Lv-ITGB1 groups, the ITGB1 overexpression lentivirus and its negative control lentivirus were transfected into SiHa cells transfected with Lv-KLF14. Flow cytometry confirmed that when ITGB1 was solely overexpressed (Lv-ITGB1), the apoptosis rate of cervical cancer SiHa cells was lower than that of the Lv-control (ITGB1) group, indicating that ITGB1 inhibits the apoptosis of cervical cancer cells. However, when ITGB1 and KLF14 constructs were co transfected, the above effect was reversed, and the apoptosis rate of cervical cancer cells overexpressing both ITGB1 and KLF14 (Lv-ITGB1 + Lv-KLF14) was higher than that of the Lv-control (KLF14) + Lv-ITGB1 group. In addition, the apoptosis rate of cervical cancer cells treated with Lv-KLF14 + Lv-ITGB1 was lower than that of cervical cancer cells treated with Lv-KLF14 + Lv-control (ITGB1) (Fig. 5C). These results indicated that KLF14 could regulate ITGB1, and KLF14 promoted apoptosis and reversed the effect of ITGB1 on the apoptosis of cervical cancer cells. Moreover, ITGB1 inhibited apoptosis and reversed the effect of KLF14 on cervical cancer cell apoptosis.

### KLF14 regulates related molecules in the PI3K/AKT signalling pathway

**Fig. 6:**
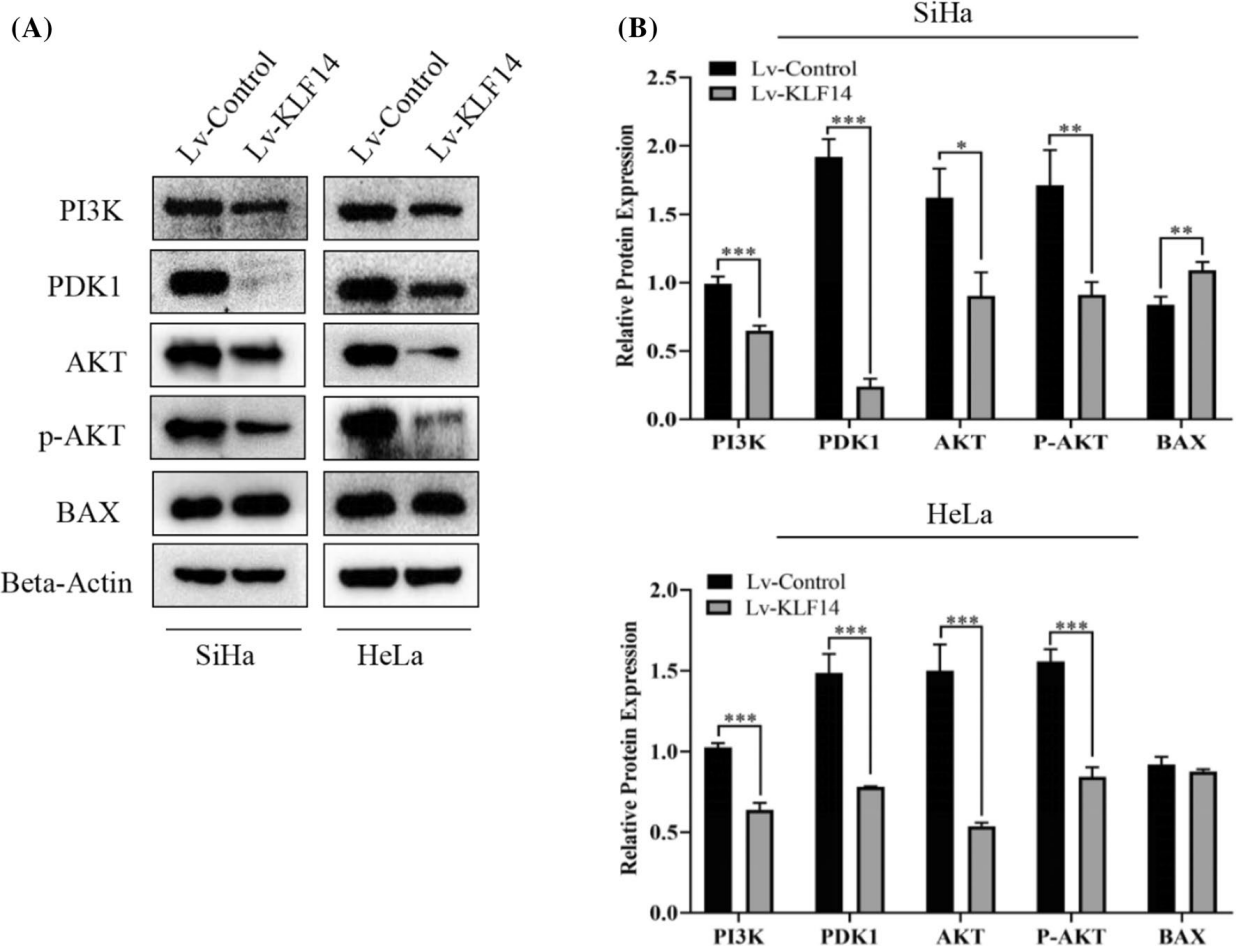
KLF14 regulates related molecules in the PI3K/AKT signalling pathway. **A** When KLF14 was overexpressed, the Western blot results showed that PI3K, PDK1, AKT and p-AKT were downregulated and Bax was upregulated in SiHa cells compared to the Lv-control group cells. PI3K, PDK1, AKT and p-AKT were downregulated, and Bax showed no significant difference in HeLa cells compared to the Lv-control group cells. **B** Quantitative statistics of protein expression. (*P < 0.05, **P < 0.01, ***P < 0.001)

To understand the underlying mechanism, we carried out further experiments. The Western blot results showed that PI3K, PDK1, AKT and p-AKT were downregulated when KLF14 was overexpressed in both SiHa and HeLa cells. Compared with the Lv-control group, the expression of Bax in SiHa cells was upregulated in the Lv-KLF14 group, while the expression of Bax in HeLa cells showed no significant difference between the Lv-control group and Lv-KLF14 group. Therefore, we believe that KLF14 may regulate related molecules in the PI3K/AKT signalling pathway (Fig. 6A, B).

### KLF14 targets ITGB1 and inhibits the development of cervical cancer through the PI3K/AKT signalling pathway

**Fig. 7:**
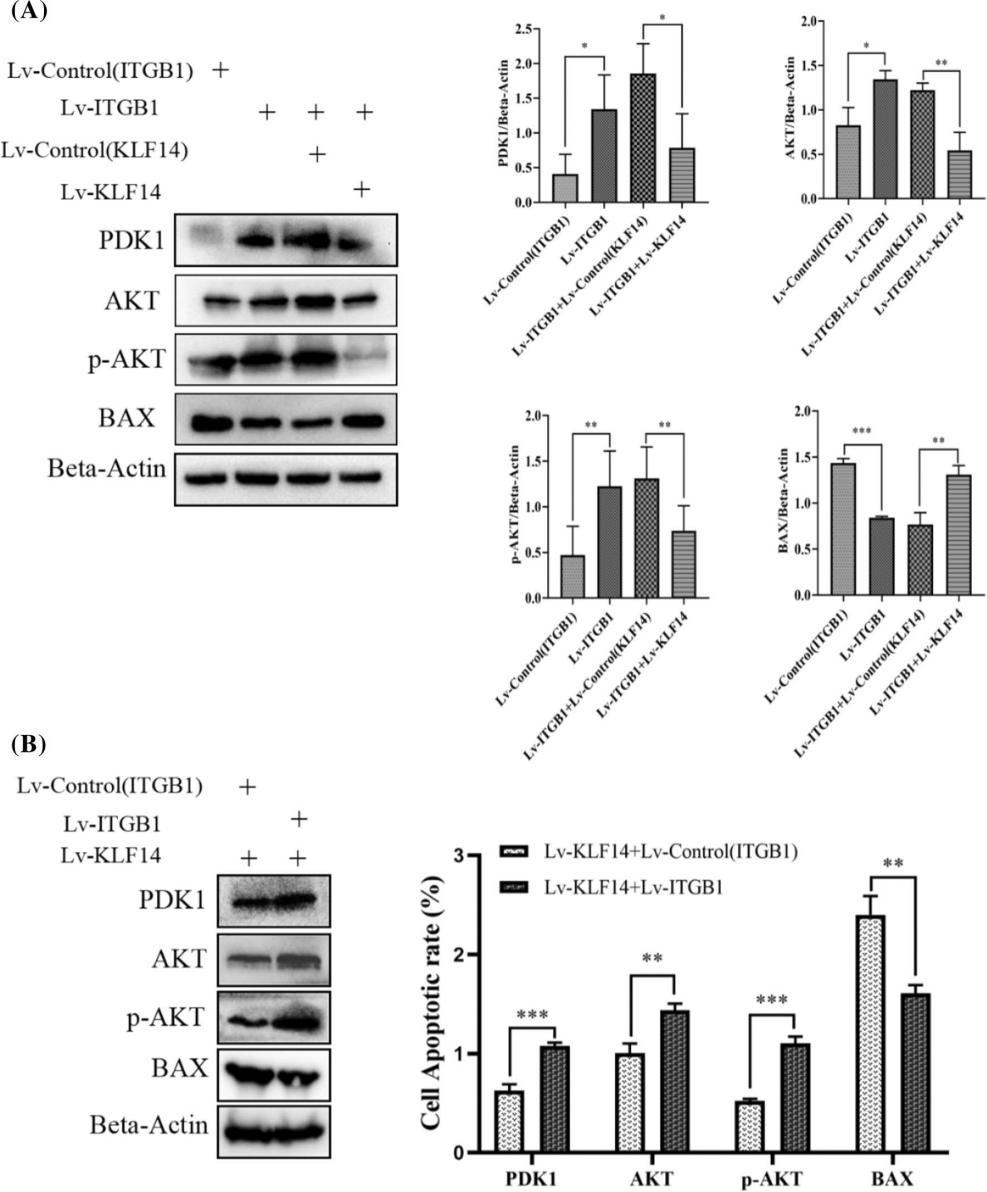
KLF14 targets ITGB1 and inhibits the development of cervical cancer through the PI3K/AKT signalling pathway. **A** Four groups were established: Lv-control (ITGB1), Lv-ITGB1, Lv-ITGB1 + Lv-control (KLF14) and Lv-ITGB1 + Lv-KLF14 (the KLF14 overexpression lentivirus and its negative control lentivirus were transfected into SiHa cells transfected with Lv-ITGB1). When ITGB1 was overexpressed, compared to the Lv-control (ITGB1) group, the Western blot results showed that the expression of PDK1, AKT and p-AKT was upregulated, while Bax was downregulated. However, when Lv-KLF14 and Lv-ITGB1 were cotransfected, the expression of PDK1, AKT and p-AKT was downregulated compared to that in the Lv-ITGB1 + Lv-control (KLF14) group, while Bax was upregulated. B. Two groups were established: Lv-KLF14 + Lv-control (ITGB1) and Lv-KLF14 + Lv-ITGB1 (the ITGB1 overexpression lentivirus and its negative control lentivirus were transfected into SiHa cells transfected with Lv-KLF14). For Lv-KLF14 + Lv-control (ITGB1) and Lv-KLF14 + Lv-ITGB1, the Western blot results showed that the expression of PDK1, AKT and p-AKT in the Lv-KLF14 + Lv-ITGB1 group was upregulated compared to that in the Lv-KLF14 + Lv-control (ITGB1) group, while Bax was downregulated. (*P < 0.05, **P < 0.01, ***P < 0.001)

SiHa cells were used for the rescue experiments. First, four groups were established: Lv-control (ITGB1), Lv-ITGB1, Lv-ITGB1 + Lv-control (KLF14) and Lv-ITGB1 + Lv-KLF14 (the KLF14 overexpression lentivirus and its negative control lentivirus were transfected into SiHa cells transfected with Lv-ITGB1). The Western blot results showed that when ITGB1 was solely overexpressed, the expression of PDK1 and AKT was upregulated compared to that in the Lv-control (ITGB1) group and the level of p-AKT was increased, while Bax was downregulated. However, when Lv-KLF14 and Lv-ITGB1 constructs were cotransfected, the above phenomenon was reversed. Compared to that in the Lv-ITGB1 + Lv-control (KLF14) group, the expression of PDK1 and AKT in the cotransfection group was downregulated, and the level of p-AKT was decreased, while Bax was upregulated. This result implied that the effect of ITGB1 on the related molecules in the PI3K/AKT pathway in cervical cancer cells could be rescued by KLF14 (Fig. 7A). Then, two groups were established: Lv-KLF14 + Lv-control (ITGB1) and Lv-KLF14 + Lv-ITGB1 (the ITGB1 overexpression lentivirus and its negative control lentivirus were transfected into SiHa cells transfected with Lv-KLF14). The Western blot results showed that the expression of PDK1 and AKT in Lv-KLF14 + Lv-ITGB1 was upregulated and the level of p-AKT was increased and Bax was downregulated compared to that in Lv-KLF14 + Lv-control (ITGB1). The presence of p-AKT indicates the activation of the PI3K/AKT signalling pathway in response to a particular stimulus. This finding suggested that the effect of KLF14 on the related molecules in the PI3K/AKT pathway in cervical cancer cells could be rescued by ITGB1 (Fig. 7B). Therefore, we believe that KLF14 and ITGB1 may be correlated and affect the development of cervical cancer through the PI3K/AKT signalling pathway. Schematic graph was drawn and attached to Additional file 3.

## Discussion

Cervical cancer is one of the most common malignancies in women worldwide [6]. Various factors lead to the occurrence of cervical cancer. Although the development of HPV vaccines has prevented cervical cancer to some extent, the incidence and mortality rates of cervical cancer are still high [25]. We aimed to find more effective biomarkers for the diagnosis and treatment of cervical cancer. KLF14 plays an important role in a variety of physiological and pathological processes. In recent years, the number of studies exploring the relationship between KLF14 and cancer has increased. Zhou et al. found that the lncRNA HAND2-AS1 inhibits the progression of colon cancer by regulating the expression of KLF14 [26]. Luo et al. demonstrated that KLF14 can regulate the antioxidant response by regulating the HO-1 pathway in castrate-resistant prostate cancer, thus providing a treatment target for castrate-resistant prostate cancer patients [27]. In addition, KLF14 has been reported to be associated with HCC and breast cancer [14, 28].

In this study, we explored whether KLF14 is also associated with cervical cancer. First, at the tissue level, we used human female cervical cancer tissue and paracancerous tissue chips for validation. Immunohistochemical assays confirmed that the expression of KLF14 in cervical cancer tissues was lower than that in paracancerous tissues. Then, at the cellular level, we verified that the proliferation of KLF14-overexpressing cervical cancer cells was inhibited according to CCK-8 and colony formation assays. To verify the effect of KLF14 on the progression of cervical cancer in vivo, we used SiHa cell suspensions from the Lv-KLF14 group as a positive control and the Lv-control group as a negative control to construct a subcutaneous neoplasia model in nude mice. In this experiment, DOX played an important role as an inducer of KLF14 expression, which could induce KLF14 expression in the Lv-KLF14 group, thus creating a control between the Lv-KLF14 group and the Lv-control group without KLF14 expression. In vivo results confirmed that subcutaneous tumours in the KLF14-overexpression group were smaller, lower in weight, and grew more slowly than those in the Lv-control group. These results indicated that KLF14 can inhibit the development of cervical cancer in vivo. In addition, flow cytometry showed that KLF14-overexpressing cervical cancer cells had a higher rate of apoptosis than negative control cells, which confirmed the role of KLF14 in promoting apoptosis of cervical cancer cells. In summary, these results suggest that KLF14 plays an inhibitory role in the progression of cervical cancer and may be helpful in the treatment of cervical cancer in the future.

Recently, there has been increased research on the relationship between ITGB1, a member of the ITG family, and tumours. Min et al. found that ITGB1 overexpression can regulate the Notch signalling pathway and thus significantly promote the proliferation of glioma cells [29]. Liang et al. confirmed that ITGB1 is a target of miR-493-5p and that NSCLC patients with high expression of ITGB1 and low expression of miR-493-5p have a shorter survival period and poorer prognosis than those with other expression patterns [30]. Guo et al. showed that THBS4 can interact with ITGB1 and activate the downstream PI3K/AKT pathway, thereby promoting the proliferation and metastasis of HCC [31].

In this study, we considered whether there might be some association between KLF14 and ITGB1. First, Western blotting was performed to verify that when KLF14 was overexpressed, ITGB1 expression was downregulated in cervical cancer SiHa and HeLa cells. This revealed that KLF14 could inhibit ITGB1 expression in cervical cancer cells at the protein level. Second, regulation at the transcription level is an important link in the regulation of gene expression. Since KLF14 is a transcription factor, we considered whether KLF14 affects the transcription of ITGB1 and thus regulates its expression level. We confirmed that KLF14 downregulated ITGB1 expression using a dual-luciferase reporter assay. This finding indicated that KLF14 can directly target ITGB1 and inhibit ITGB1 expression at the transcriptional level. Third, to further prove the relationship between KLF14 and ITGB1 and their effects on cervical cancer, we conducted functional experiments. We demonstrated the role of KLF14 in promoting apoptosis of cervical cancer cells. Flow cytometry experiments confirmed that the apoptosis rate of SiHa cells decreased when ITGB1 was overexpressed, indicating that ITGB1 inhibited the apoptosis of cervical cancer cells. The effect of ITGB1 on cervical cancer cell apoptosis was reversed by KLF14, resulting in increased apoptosis. We also found that ITGB1 reversed the effect of KLF14 on cervical cancer cell apoptosis, resulting in decreased apoptosis. These results further proved the association between KLF14 and ITGB1 at the functional level, as well as the effect on apoptosis of cervical cancer cells. Furthermore, in the in vivo experiment, we detected the expression of relevant molecules in tumour tissues, including KLF14, ITGB1, p-AKT (a key molecule in the PI3K/AKT signalling pathway), and cleaved caspase-3 (an apoptosis marker). Compared with the negative control group, KLF14 expression was higher in the positive group, while ITGB1 expression was downregulated, which also explained the regulatory effect of KLF14 on ITGB1 to some extent.

ITGB1 is closely correlated with the PI3K/AKT signalling pathway, and many studies have confirmed the relationship between ITGB1 and the PI3K/AKT signalling pathway [19, 32]. We considered whether the effect of KLF14 targeting ITGB1 on the progression of cervical cancer was related to the PI3K/AKT signalling pathway. Subsequently, we investigated the mechanism in greater depth. We used Western blotting to verify that KLF14 could affect related molecules in the PI3K/AKT pathway, including PI3K, PDK1, AKT, p-AKT and Bax. ITGB1 could also affect related molecules in the PI3K/AKT signalling pathway; PDK1, AKT and p-AKT were upregulated, and Bax was downregulated when ITGB1 was overexpressed. Meanwhile, we found that ITGB1 also regulates related molecules in the PI3K/AKT signaling pathway, including PDK1, AKT, P-AKT, and BAX. These molecules are closely associated with apoptosis, and AKT in particular, as a key molecule in this pathway, plays a pivotal role in inhibiting the production of pro-apoptotic signals or inhibiting pro-apoptotic proteins. This may provide evidence for our finding that ITGB1 inhibits apoptosis of cervical cancer cells confirmed by flow cytometry. In addition, the effect of ITGB1 on downstream related molecules could be reversed by KLF14, and the effect of KLF14 on downstream related molecules could also be reversed by ITGB1. The above results indicated that KLF14 targeting ITGB1 affects the progression of cervical cancer via the PI3K/AKT signalling pathway. In addition, very interestingly, one study found that autocrine and paracrine phosphorylation of STAT3 in HPV-positive cervical cancer cells are driven by activation of the IL-6 signaling axis, thus promoting the proliferation of cervical cancer cells. This study suggests that targeted regulation of this signaling pathway may provide new ideas for the treatment of HPV positive cervical cancer [33]. In our study, we found a relationship between the PI3K/AKT signaling pathway and cervical cancer. We considered that the proliferation of cervical cancer cells may be associated with the activation of the PI3K/AKT signaling pathway, so the relationship between the PI3K/AKT pathway, KLF14 and HPV-positive cervical cancer is worthy of further study.

Inevitably, there are many shortcomings related to our research. First, the KLF14 expression levels of cervical cancer cells were not compared with those of normal cervical cells due to the difficulty in obtaining normal cervical cells. Second, the number of cases used for immunohistochemistry analysis was limited, and an assessment of more cases would provide stronger evidence. Third, the rescue experiments were only performed in SiHa cervical cancer cells, which may not be a comprehensive approach because different cells have distinct characteristics and may show different experimental phenomena. In addition, we demonstrated that KLF14 targets ITGB1, but a more explicit and deeper relationship between these molecules is still worth exploring. These limitations need to be addressed in the future.

## Conclusion

In conclusion, this study confirmed that KLF14 inhibits the proliferation and promotes the apoptosis of cervical cancer cells. ITGB1 also plays an important role in this process, as KLF14 inhibits the progression of cervical cancer by targeting ITGB1 through the PI3K/AKT signalling pathway. KLF14 may be a potential and noteworthy target in the treatment of cervical cancer.

## Supplementary Information

Additional file 1: Specific patient information in the tissue microarray. (PDF 41 KB)

Additional file 2: Treatment scheme of in vivo experiment, including injection scheme and induction scheme. (PDF 176 KB)

Additional file 3: Schematic graph. (TIF 10800 KB)

Additional file 4 (PDF 83 KB)

## Data Availability

All data generated or analysed in this study are available in the article.

## Abbreviations

KLF14: Krüppel-like factor 14
ITGB1: Integrin β1
IHC: Immunohistochemistry
TNM: Tumour-node-metastasis
CCK-8: Cell counting kit-8
HPV: Human papillomavirus
HCC: Hepatocellular carcinoma
ITG: Integrin
DOX: Doxorubicin
p-AKT: Phospho-AKT
PI3K: Phosphoinositide-3 kinase
