# Supplementary material for: KLF14 targets ITGB1 to inhibit the progression of cervical cancer via the PI3K/AKT signalling pathway
Source: Discov Oncol. 2022 May 16;13:30. doi: 10.1007/s12672-022-00494-1 (PMC9108130; doi:10.1007/s12672-022-00494-1)
Supplement: Supplementary file 1 — Additional file 1: Specific patient information in the tissue microarray. (PDF 41 KB) [file 12672_2022_494_MOESM1_ESM.pdf]

Patient	Operation Organ	Sex	Age	Pathological Type	T	N	M	Clinical Stage
J03A0309	uterus	female	46	squamous cell carcinoma	T1a1	N0	M0	IA1
J03A0306	uterus	female	43	squamous cell carcinoma	T1a2	N0	M0	IA2
J03A0215	uterus	female	adult	squamous cell carcinoma	T1b1	N0	M0	IB1
J03A0216	uterus	female	adult	squamous cell carcinoma	T1b1	N0	M0	IB1
J03A0223	uterus	female	adult	squamous cell carcinoma	T1b1	N0	M0	IB1
J03A0224	uterus	female	adult	squamous cell carcinoma	T1b1	N0	M0	IB1
J03A0225	uterus	female	adult	squamous cell carcinoma	T1b1	N0	M0	IB1
J03A0229	uterus	female	adult	squamous cell carcinoma	T1b1	N0	M0	IB1
J03A0230	uterus	female	adult	squamous cell carcinoma	T1b1	N0	M0	IB1
J03A0234	uterus	female	adult	squamous cell carcinoma	T1b1	N0	M0	IB1
J03A0236	uterus	female	adult	squamous cell carcinoma	T1b1	N0	M0	IB1
J03A0237	uterus	female	adult	squamous cell carcinoma	T1b1	N0	M0	IB1
J03A0238	uterus	female	adult	squamous cell carcinoma	T1b1	N0	M0	IB1
J03A0239	uterus	female	adult	squamous cell carcinoma	T1b1	N0	M0	IB1
J03A0241	uterus	female	adult	squamous cell carcinoma	T1b1	N0	M0	IB1
J03A0164	uterus	female	54	squamous cell carcinoma	T1b1	N0	M0	IB1
J03A0180	uterus	female	54	squamous cell carcinoma	T1b1	N0	M0	IB1
J03A0031	uterus	female	60	squamous cell carcinoma	T1b1	N0	M0	IB1
J03A0077	uterus	female	56	squamous cell carcinoma	T1b1	N0	M0	IB1
J03A0366	uterus	female	52	squamous cell carcinoma	T1b1	N0	M0	IB1
J03A0028	uterus	female	65	squamous cell carcinoma	T1b2	N0	M0	IB2
J03A0040	uterus	female	48	squamous cell carcinoma	T1b2	N0	M0	IB2
J03A0105	uterus	female	57	squamous cell carcinoma	T1b2	N0	M0	IB2
J03A0039	uterus	female	29	squamous cell carcinoma	T1b	N0	M0	IB
J03A0227	uterus	female	adult	squamous cell carcinoma	T1b	N0	M0	IB
J03A0041	uterus	female	36	squamous cell carcinoma	T1b	N0	M0	IB
J03A0067	uterus	female	70	squamous cell carcinoma	T1b	N0	M0	IB
J03A0074	uterus	female	38	squamous cell carcinoma	T1b	N0	M0	IB
J03A0075	uterus	female	52	squamous cell carcinoma	T1b	N0	M0	IB
J03A0085	uterus	female	61	squamous cell carcinoma	T1b	N0	M0	IB
J03A0098	uterus	female	45	squamous cell carcinoma	T1b	N0	M0	IB
