# Supplementary material for: KLF14 targets ITGB1 to inhibit the progression of cervical cancer via the PI3K/AKT signalling pathway
Source: Discov Oncol. 2022 May 16;13:30. doi: 10.1007/s12672-022-00494-1 (PMC9108130; doi:10.1007/s12672-022-00494-1)
Supplement: Supplementary file 2 — Additional file 2: Treatment scheme of in vivo experiment, including injection scheme and induction scheme. (PDF 176 KB) [file 12672_2022_494_MOESM2_ESM.pdf]

Injection Scheme

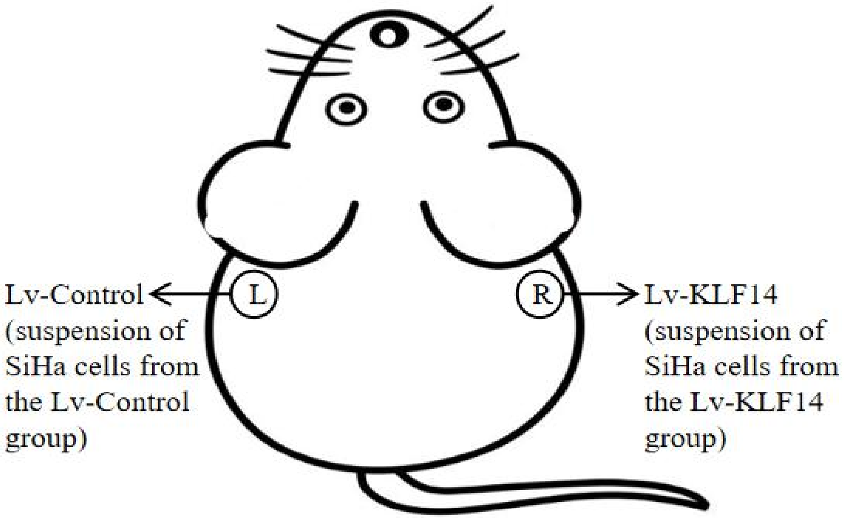

Induction Scheme

TIME	METHOD DESIGN	PURPOSE
Before the injection	SiHa cells (Lv-KLF14 group and Lv-control group) were cultured in medium containing DOX （5ug/ml）for 48 h	In vitro induction of KLF14 expression
Within one week of injection	Dox-containing water（1mg/ml） was fed continuously.	In vivo induction of KLF14 expression
After one week of injection	Dox-containing water（1mg/ml）was fed intermittently (Reduces the damage of DOX toxicity to animals).	In vivo induction of KLF14 expression
