# Supplementary figures and images for: KLF14 targets ITGB1 to inhibit the progression of cervical cancer via the PI3K/AKT signalling pathway
Source: Discov Oncol. 2022 May 16;13:30. doi: 10.1007/s12672-022-00494-1 (PMC9108130; doi:10.1007/s12672-022-00494-1)

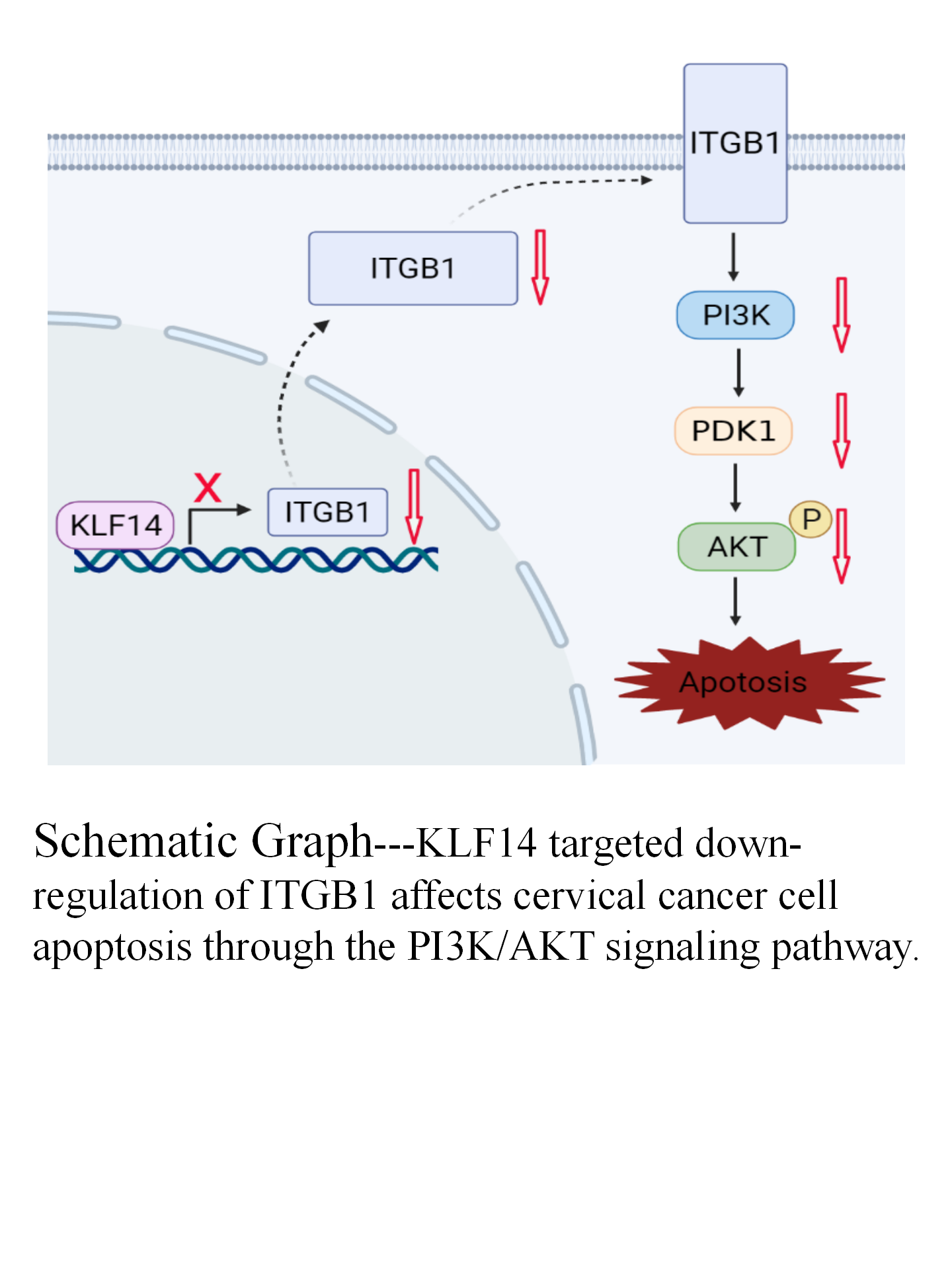

Supplement: Supplementary file 3 — Additional file 3: Schematic graph. (TIF 10800 KB) [file 12672_2022_494_MOESM3_ESM.tif]
