# Supplementary material for: KLF14 targets ITGB1 to inhibit the progression of cervical cancer via the PI3K/AKT signalling pathway
Source: Discov Oncol. 2022 May 16;13:30. doi: 10.1007/s12672-022-00494-1 (PMC9108130; doi:10.1007/s12672-022-00494-1)
Supplement: Supplementary file 4 — Additional file 4 (PDF 83 KB) [file 12672_2022_494_MOESM4_ESM.pdf]

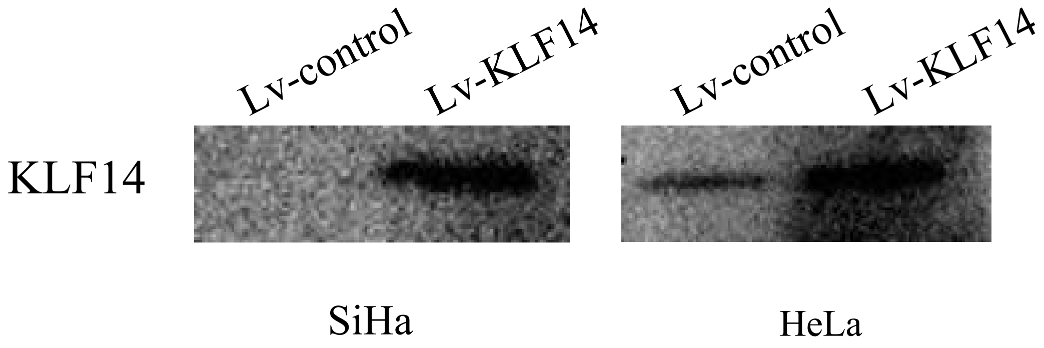

We transfected cervical cancer cells with KLF14-overexpressing lentivirus and its negative control lentivirus, and detected the expression level of KLF14 with KLF14 antibody(Sigma-Aldrich, HPA044729). The Lv-control group can be regarded as primitive cells and can represent endogenous levels of KLF14 expression. The Lv-KLF14 group was compared with the Lv-control group. The result is shown below.
